# Arc-Fault Detection Using Stage-Wise Alignment and Feature Fusion of Dual Learnable Time–Frequency Representations

**DOI:** 10.3390/s26165095

**Published:** 2026-08-11

**Authors:** Seoyoung Jeon, Won-Kyu Choi, Sungsoo Kwon, Jonghyuk Lee, Ji-Hoon Bae

**Affiliations:** 1 Department of AI and Big Data Engineering, Daegu Catholic University, Gyeongsan 38430, Republic of Korea; jeon2002sy@cu.ac.kr (S.J.); jonghyuk@cu.ac.kr (J.L.); 2 Electronics and Telecommunications Research Institute, Daejeon 34129, Republic of Korea; wkchoi@etri.re.kr; 3 Corporate R&D Center, MongooseAI Co., Ltd., Seongnam 13486, Republic of Korea; kwonss@mongooseai.com; 4 Department of Computer Education, Korea National University of Education, Cheongju 28173, Republic of Korea

**Keywords:** arc-fault detection, current waveform analysis, feature fusion, learnable time–frequency representation, smart-farm distribution system, representation alignment

## Abstract

Arc faults in low-voltage smart-farm distribution systems are difficult to detect reliably because normal current waveforms vary with load composition and operating conditions. This paper presents a progressive three-stage time–frequency learning framework that identifies series arc faults directly from normalized current waveforms. In Stage I, fast Fourier transform (FFT)-based and wavelet-based learnable front-ends independently learn complementary global spectral and local transient representations. In Stage II, their representation spaces are structured using supervised contrastive learning with view-level and cross-spectrum alignment. In Stage III, the aligned feature extractors are frozen, and only the feature fusion block and final classifier are trained for 10-class classification. Current signals are acquired from a practical smart-farm distribution system and evaluated using a measurement-session-level group-wise split. The framework achieves 99.92% accuracy and 99.93% macro recall while maintaining 1.06 M parameters and a model-only inference latency of 8.70 ms. The results demonstrate that the proposed framework provides robust discrimination across unseen measurement sessions within the evaluated load categories and operating conditions.

## 1. Introduction

Electrical arc faults are critical failure events in low-voltage distribution systems because they can produce irregular current distortion, localized heating, insulation degradation, and electrical fires. In practical electrical facilities, early arc-fault detection is essential for preventing safety accidents and maintaining reliable operation. This requirement is particularly important in smart-farm environments, where electrical equipment operates continuously to control ventilation, lighting, heating, and other environmental conditions [1,2].

Arc-fault detection in smart-farm distribution systems is challenging because normal current waveforms vary substantially according to load composition and operating conditions. Loads such as ventilation fans, fluorescent lamps, heating lamps, incandescent lamps, and light-emitting diode (LED) lamps exhibit different waveform patterns even under normal operation. When these loads are connected individually to the same feeder, load-dependent current variations can partially overlap with fault-induced disturbances. Therefore, a detection model must capture fault-consistent patterns while remaining robust to normal waveform variability caused by heterogeneous loads.

Conventional arc-fault detection methods have commonly used threshold rules, harmonic indicators, handcrafted statistical features, and fixed time–frequency transforms such as the fast Fourier transform (FFT), short-time Fourier transform (STFT), and wavelet transform [3,4,5,6]. These approaches provide useful information about abnormal frequency components, energy distribution, and transient waveform distortion. Related high-resolution waveform studies have also investigated transient and incipient events in power distribution networks using synchronized voltage and current measurements for event detection, classification, and fault-location analysis [7,8,9]. These studies further highlight the value of detailed waveform signatures for characterizing short-duration and intermittently recurring electrical disturbances, which is also relevant to the waveform behavior of series arcing. However, their feature sets or transform parameters are generally determined in advance, which limits their adaptability when load types, operating conditions, or noise characteristics change. Deep learning methods reduce the dependence on manual feature design by learning representations from current waveforms, but a single representation may not sufficiently describe both global spectral characteristics and localized transient patterns [10,11].

To address these limitations, this paper presents a progressive three-stage time–frequency learning framework for arc-fault detection in low-voltage smart-farm distribution systems. In Stage I, an FFT-based learnable spectral front-end and a learnable wavelet front-end independently learn complementary representations from normalized current waveforms. The FFT-based branch captures global spectral characteristics, whereas the wavelet-based branch captures localized multi-scale transient patterns. In Stage II, the two representation spaces are structured using supervised contrastive learning with view-level and cross-spectrum alignment objectives. In Stage III, the aligned feature extractors are frozen, and only the feature fusion block and final classifier are optimized for 10-class classification.

The main contributions of this study are summarized as follows. First, a dual-front-end architecture is designed to learn complementary spectral and transient representations from raw current waveforms using only segment-wise normalization, without handcrafted feature extraction. Second, a progressive alignment procedure is introduced to organize the learned time–frequency representations before final fusion using supervised contrastive, view-level, and cross-spectrum objectives. Third, the proposed framework is evaluated using current signals acquired from a practical smart-farm distribution system with a data-leakage-aware measurement-session-level split. The experimental results show high arc-fault detection performance, compact model complexity, and low model-only inference latency.

## 2. Data Acquisition and Problem Setup

This section describes the current-signal dataset used for arc-fault detection and defines the classification problem considered in this study. The measurement setup, load conditions, sliding-window segmentation, class definition, and measurement-session-level data split are presented.

### 2.1. Measurement Setup

Current signals were acquired from a practical low-voltage smart-farm distribution system. The measurement environment included representative farm loads, such as ventilation fans, fluorescent lamps, heating lamps, incandescent lamps, and light-emitting diode (LED) lamps. These loads were connected individually to a single distribution feeder to reflect load-dependent waveform variability under practical operating conditions.

Each tested load was connected in series with a UL 1699-compliant arc-fault generator under a 220 V/60 Hz AC supply. Intermittent series arcing was produced by periodically adjusting the gap between a carbon electrode and a copper electrode [12,13].

**Figure 1:**
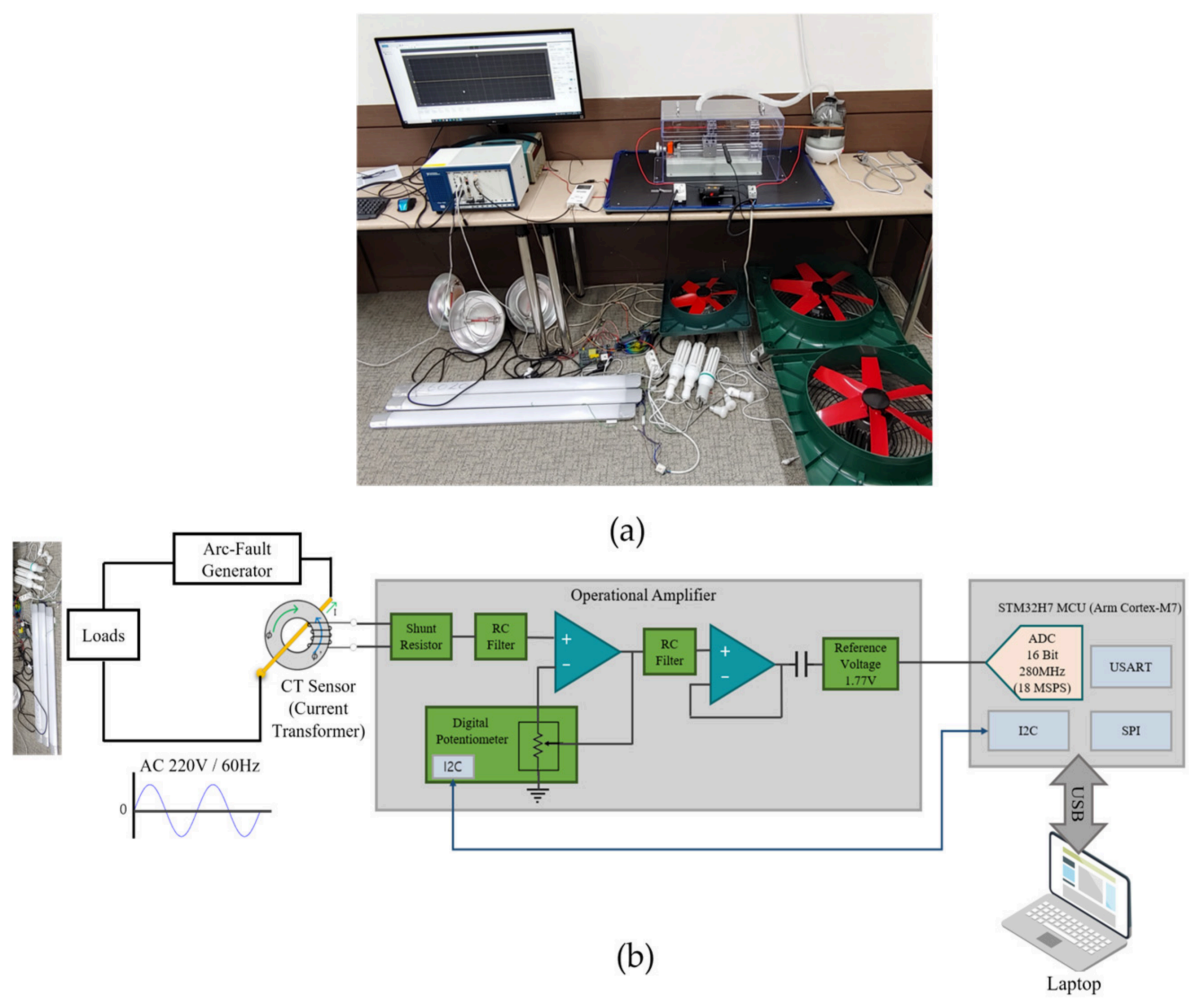
Experimental setup and current-signal acquisition system: (**a**) photograph of the actual experimental setup and representative test loads; (**b**) single-line diagram of the 220 V/60 Hz series arc-fault circuit and the CT-based signal-conditioning and data-acquisition chain.

The resulting branch current was measured using a current transformer (CT). The CT output was converted into a voltage signal using a 10 Ω shunt resistor and processed by RC filters and a TLC2272CDR-based variable-gain amplifier with a gain range of 0–47.9 dB. The conditioned signal was level-shifted using a 1.77 V reference voltage and digitized at 1 MS/s using the 16-bit analog-to-digital converter (ADC) integrated into an STM32H7A3RGT6 microcontroller. For clarity, the sampling rate used to acquire the dataset analyzed in this study was 1 MS/s; the 18 MSPS label shown in Figure 1b does not represent the acquisition rate used in these experiments. The acquired data were transferred to a PC through a USB interface, and approximately 0.9 s of each continuous acquisition record was used for sliding-window segmentation.

Current sensing was selected because the intermittent conduction and waveform distortions caused by a series arc are directly reflected in the branch current. Although voltage measurements may provide complementary information, the visibility of voltage signatures depends on the measurement point relative to the fault, whose location is generally unknown in practical low-voltage distribution systems. A single CT-based channel therefore provides a simple and practical configuration for monitoring low-voltage distribution circuits.

Figure 1 presents the experimental configuration, including a photograph of the setup and representative loads in Figure 1a and a single-line diagram of the series arc-fault circuit and CT-based signal acquisition chain in Figure 1b.

### 2.2. Segmentation and Class Definition

For each load condition, both normal operation and series arc-fault operation were repeatedly measured under the same acquisition settings. After dataset construction, the final dataset consisted of 522,340 segmented current waveform samples generated from 1066 measurement sessions. The dataset contains 10 load-state classes, corresponding to the combinations of five load types and two operating states: normal and arc fault. In this study, a measurement session denotes one continuous current waveform acquisition under a specific load type and operating state, a segmented waveform sample denotes one model input generated through sliding-window segmentation, and a sampled data point denotes one digitized current value contained within a segmented waveform sample. The class-wise distributions of segmented waveform samples across the training, validation, and test sets are summarized in Table 1.

The digitized current signals were used without additional digital filtering, downsampling, denoising, or handcrafted feature extraction. Each segmented waveform sample was generated using a fixed sliding window of 18,000 sampled data points, corresponding to an observation interval of 18 ms at a sampling rate of 1 MS/s. The stride was set to 1800 sampled data points, corresponding to 1.8 ms; therefore, adjacent segmented waveform samples had an overlap ratio of 90.0%. Because one cycle of a 60 Hz waveform corresponds to approximately 16.67 ms, the 18 ms window includes one complete cycle together with a short interval near the adjacent cycle boundary. This allows each input segment to contain zero-crossing characteristics, half-cycle discontinuities, periodic high-frequency components within a cycle, and short-term waveform variations near the cycle boundary. Using these segmentation settings, 490 segmented waveform samples were generated from each measurement session. Across 1066 measurement sessions, this procedure produced a total of 522,340 segmented waveform samples. Each segmented waveform sample was represented as a one-dimensional current waveform with 18,000 time steps and one channel. The dataset construction, segmentation settings, and data-split statistics are summarized in Table 2.

Each input segment is represented as a single-channel time-series vector,

(1) $$x_{i}={\left[x_{i,1},\;x_{i,2},\;\ldots ,\;x_{i,T}\right]}^{⊤}\in R^{T\times 1},$$

where T = 18,000 denotes the number of sampled data points contained in each segmented waveform sample, and $x_{i,t}$ denotes the current value of the i-th segment at time index t. To stabilize training, each segment was linearly normalized to the range [−1, 1]. No manual spectral, statistical, or time-domain features were extracted before model training.

**Figure 2:**
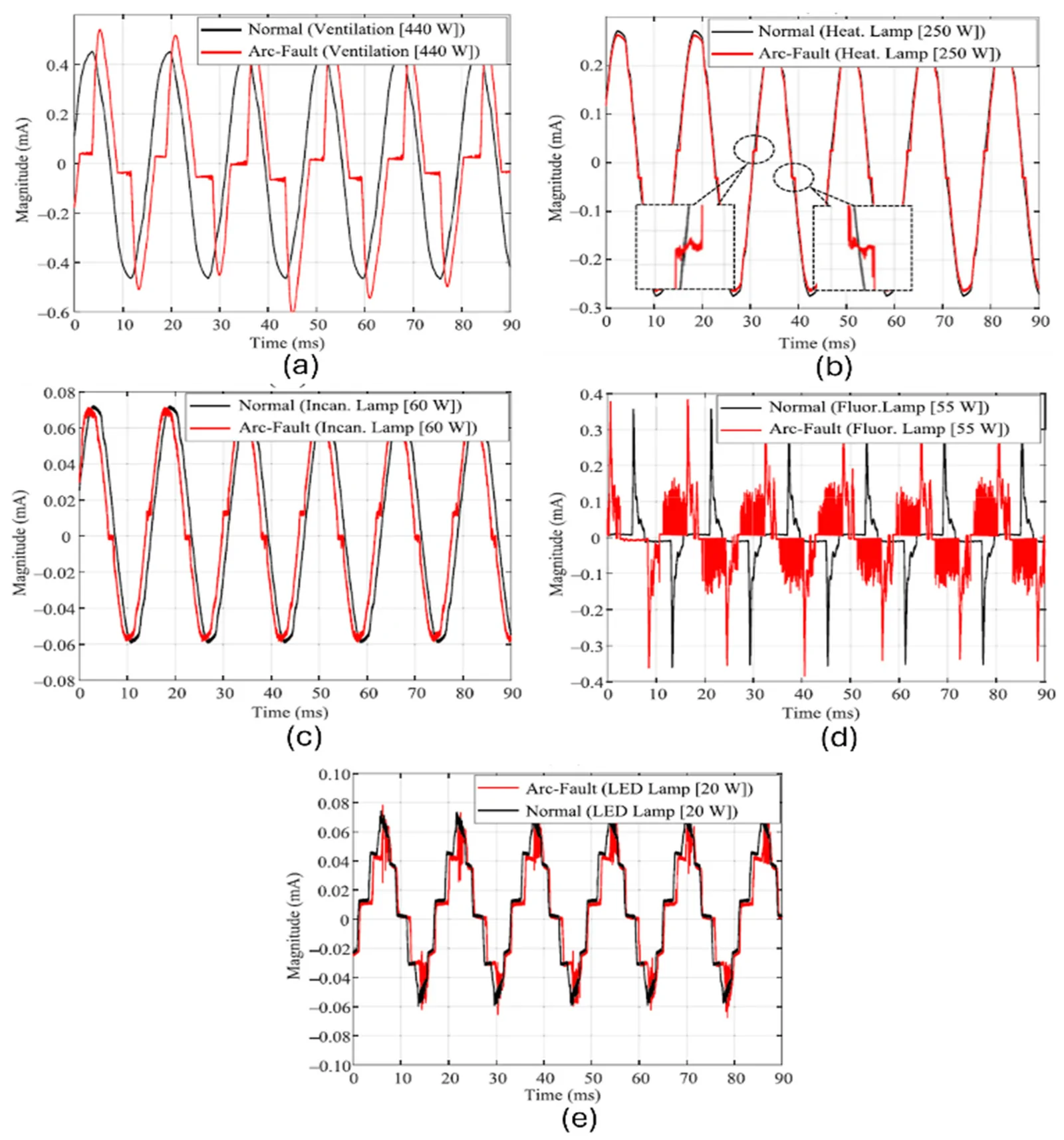
Representative current waveforms under normal and arc-fault conditions for different loads: (**a**) ventilation fan, (**b**) heating lamp, (**c**) incandescent lamp, (**d**) fluorescent lamp, and (**e**) LED lamp.

Figure 2 presents representative current waveforms under normal and arc-fault conditions for different loads. The waveform examples show that arc-fault manifestations vary depending on the load type. For example, ventilation fans and heating lamps show relatively regular waveforms under normal operation, whereas arc-fault events induce intermittent amplitude reductions and abnormal waveform distortion. Fluorescent, incandescent, and LED lamps exhibit arc-fault patterns characterized by high-frequency noise and irregular amplitude fluctuations. These observations motivate the use of representations that can capture both load-dependent waveform variability and fault-consistent signal characteristics.

The classification task was formulated as a 10-class problem corresponding to the combinations of five load categories and two operating states: normal and arc fault. During inference, the model receives only the measured current waveform, and the identity of the connected load is not provided as prior information. The predicted class therefore jointly specifies the load category and operating state, and a prediction is counted as correct only when both are correctly identified. Thus, within the five load categories evaluated in this study, the model performs joint load-state classification without requiring prior knowledge of the connected load type. This setting differs from a load-specific binary normal-versus-arc classifier in which the load identity is known in advance.

### 2.3. Group-Wise Split for Leakage Prevention

Because adjacent windows generated from the same raw waveform substantially overlap, a random sample-level split can place highly correlated segments from the same measurement session into the training, validation, and test sets. This can lead to data leakage and overly optimistic performance estimates [14,15].

To reduce this risk, each segmented sample was assigned a group ID corresponding to its original measurement session. The dataset was then divided using a measurement-session-level group-wise split. All samples sharing the same measurement-session ID were allocated exclusively to one of the training, validation, or test sets. Sliding windows were generated only within each original waveform, and no window was allowed to span across different measurement sessions.

After applying the group-wise split, the training, validation, and test sets contained 375,340, 42,140, and 104,860 samples, respectively, corresponding to 766, 86, and 214 measurement sessions. Therefore, the test set contained only windows generated from sessions that were not used during training or validation.

This protocol evaluates the model on measurement sessions that are independent from those used for training and validation. Thus, the reported performance reflects generalization to unseen acquisition sessions rather than random window-level recognition within the same waveform record.

## 3. Proposed Staged Time–Frequency Learning Framework

**Figure 3:**
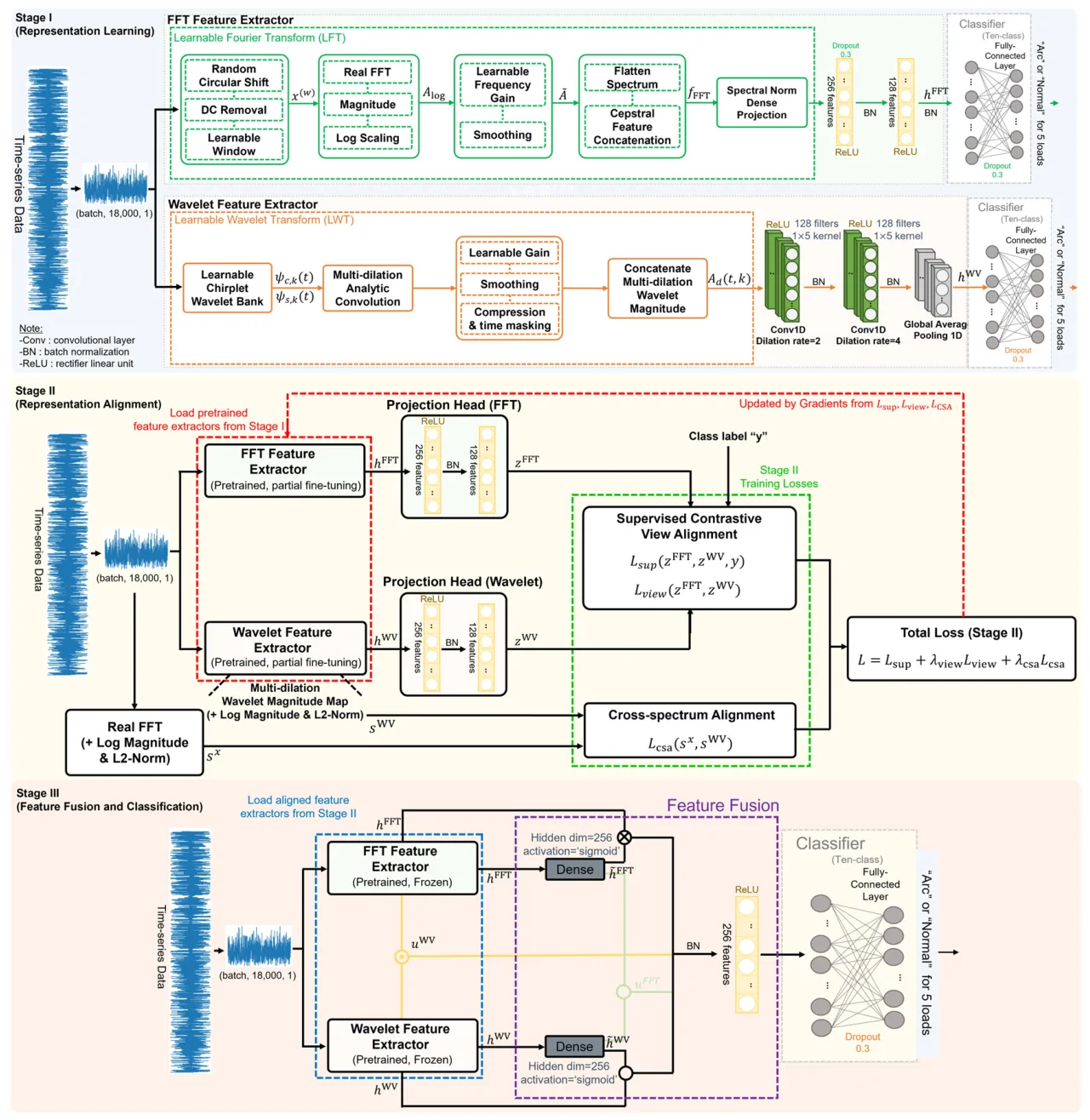
Proposed staged FFT–wavelet-based multi-view architecture with representation alignment and feature fusion using frozen aligned extractors for arc-fault classification.

This section presents the proposed progressive three-stage framework for arc-fault classification. The framework first learns spectral and transient current patterns through separate front-ends, then aligns the two representation spaces, and finally trains a fusion classifier while keeping the aligned feature extractors frozen. The overall architecture of the proposed framework is shown in Figure 3.

### 3.1. Overall Progressive Architecture

The proposed framework consists of three sequential stages: Stage I, independent front-end representation learning; Stage II, representation alignment; and Stage III, feature fusion and classification.

In Stage I, the normalized current input is fed into two parallel front-ends: the learnable Fourier transform (LFT) module and the learnable wavelet transform (LWT) module. The LFT branch produces the FFT-based feature $h^{FFT}$, which summarizes global spectral distribution patterns. The LWT branch produces the wavelet-based feature $h^{WV}$, which captures localized transient and multi-scale responses. Each branch is trained with an independent lightweight 10-class classifier so that the two front-ends can learn discriminative representations from different signal-analysis perspectives.

In Stage II, the pretrained LFT and LWT feature extractors are loaded from Stage I and partially fine-tuned for representation alignment. The features $h^{FFT}$ and $h^{WV}$ are mapped to the projected embeddings $z^{FFT}$ and $z^{WV}$ through their corresponding projection heads. Supervised contrastive learning is used to form class-wise embedding structures, view-level alignment encourages consistency between the two views for the same input segment, and cross-spectrum alignment preserves consistency between the raw input spectral signature $s^{x}$ and the wavelet-derived spectral signature $s^{WV}$.

In Stage III, the aligned feature extractors are kept frozen, while the feature fusion block and final classifier are optimized. The projection heads used during Stage II are removed, and only the feature fusion block and final classifier are trained. The fusion block integrates $h^{FFT}$ and $h^{WV}$ using cross-gating, multiplicative interaction, and discrepancy terms. This final stage allows the classifier to effectively utilize the aligned spectral and transient representations while preserving the feature structures learned in the previous stages.

### 3.2. FFT-Based Learnable Spectral Front-End

The FFT-based learnable spectral front-end is built on the standard real-valued fast Fourier transform (FFT) and augments FFT-derived spectral features through DC removal, learnable windowing, log-amplitude compression, frequency-wise gain control, smoothing, cepstral summarization, and spectral-norm dense projection [16].

First, the window-wise mean is removed from the input signal as follows:

(2) $$\bar{x}=\frac{1}{T}\sum_{t=1}^{T}x_{t},\;\;\;\;\;\;\;\;\;\;\;\;{\hat{x}}_{t}=x_{t}-\bar{x}.$$

The mean-centered signal is then multiplied by a learnable window $w=[w_{1},\ldots ,w_{T}{]}^{⊤}$ as

(3) $$x_{t}^{\left(w\right)}=w_{t}{\hat{x}}_{t},\;\;\;\;\;\;\;\;\;\;\;t=1,\;\ldots ,\;T.$$

The windowed signal is transformed into the frequency domain using the real-valued FFT. Let ${\hat{X}}_{k}$ denote the resulting complex spectrum and $A_{k}=\left|{\hat{X}}_{k}\right|$ denote the amplitude spectrum. Log compression is applied to reduce the dynamic range of spectral amplitudes as follows:

(4) $$A_{log,k}=\log\left(1+A_{k}\right),\;\;\;\;\;\;\;\;k=1,\ldots ,K.$$

A learnable frequency-wise gain vector $g=[g_{1},\ldots ,g_{K}{]}^{⊤}$ is introduced to emphasize informative frequency bands. The smoothed spectrum is computed using a local neighborhood average as follows:

(5) $${\tilde{A}}_{k}=\frac{1}{\left|N(k)\right|}\sum_{m\in N(k)}g_{m}A_{log,m},\;\;\;\;\;\;k=1,\ldots ,K,$$

where N(k) denotes a local frequency neighborhood centered at bin k. This operation allows the model to emphasize task-relevant spectral regions while reducing local spectral noise.

To summarize the spectral envelope, cepstral coefficients are extracted from the log-amplitude spectrum using the discrete cosine transform as follows:

(6) $$c_{n}=\sum_{k=1}^{K}A_{log,k}cos\left[\frac{\pi n}{K}(k-\frac{1}{2})\right],\;\;\;\;\;\;\;n=1,\ldots ,K_{c}.$$

The flattened spectrum and cepstral vector are concatenated and projected to obtain the FFT feature vector as

(7) $$f_{FFT}=\left[vec\left(\tilde{A}\right);\;c\right],\;\;h^{FFT}=\phi \left(W_{SN}f_{FFT}+b\right),$$

where vec(⋅) denotes vectorization, $W_{SN}$ denotes the spectrally normalized weight matrix, b is the bias vector, and $\phi \left(\cdot \right)$ denotes the activation function. During Stage I, a lightweight 10-class classifier is attached to $h^{FFT}$, and the LFT branch is trained independently using the cross-entropy loss. After training, the classifier head is removed, and $h^{FFT}$ is used as the spectral representation for the subsequent alignment and fusion stages.

### 3.3. Learnable Wavelet-Based Front-End

The learnable wavelet-based front-end is designed to capture localized transient patterns and multi-scale time–frequency responses. It uses a learnable analytic wavelet filter bank derived from Morlet/Chirplet-type kernels and applies multiple dilation factors through one-dimensional convolution [17,18]. This branch complements the FFT-based spectral branch by preserving local transient structures that may be weakened when the waveform is summarized only in the global frequency domain.

For filter index *k* and dilation factor d, the cosine and sine wavelet responses are computed using dilated convolution as follows:

(8) $$y_{c,k,d}\left(t\right)=\left(x*\psi_{c,k,d}\right)\left(t\right),\;{\;y}_{s,k,d}\left(t\right)=\left(x*\psi_{s,k,d}\right)\left(t\right),$$

where ∗ denotes convolution, and $\psi_{c,k,d}$ and $\psi_{s,k,d}$ denote the cosine and sine wavelet filters with dilation d, respectively.

The wavelet amplitude is then obtained from the cosine and sine responses as

(9) $$a_{k,d}\left(t\right)=\sqrt{y_{c,k,d}{\left(t\right)}^{2}+y_{s,k,d}{\left(t\right)}^{2}}.$$

A learnable gain is applied to emphasize informative scale–dilation responses, followed by smoothing and compression, as follows:

(10) $$A_{k,d}\left(t\right)=C\left(\frac{1}{\left|N(t)\right|}\sum_{\tau \in N(t)}g_{k,d}a_{k,d}(\tau )\right).$$

where N(t) denotes a local temporal neighborhood, $g_{k,d}$ denotes the learnable gain for each scale–dilation pair, and C(⋅) denotes log compression or per-channel energy normalization. The resulting multi-dilation wavelet magnitude maps are concatenated and processed using one-dimensional convolutional layers and global average pooling to produce the wavelet feature vector $h^{WV}$.

During Stage I, the LWT branch is trained with a lightweight 10-class classifier, and $h^{WV}$ is then used as the transient representation for the subsequent stages.

### 3.4. Representation Alignment

Stage II aligns the FFT and wavelet representation spaces learned in Stage I. Because the two front-ends describe the same input segment from different time–frequency perspectives, their outputs are mapped into a shared embedding space and optimized with class-level, view-level, and spectral-level consistency [19,20]. This alignment stage organizes the spectral and wavelet representations before they are used in the final fusion block.

The feature vectors $h^{FFT}$ and $h^{WV}$ are mapped to low-dimensional embeddings through projection heads as follows:

(11) $$z_{i}^{FFT}=P_{FFT}\left(h_{i}^{FFT}\right),\;\;\;\;\;z_{i}^{WV}=P_{WV}\left(h_{i}^{WV}\right),$$

where $P_{FFT}(\cdot )$ and $P_{WV}(\cdot )$ denote the projection heads for the FFT and wavelet features, respectively.

For stable similarity computation, the embeddings are $l_{2}$-normalized as

(12) $${\tilde{z}}_{i}^{FFT}=\frac{z_{i}^{FFT}}{{\left\|z_{i}^{FFT}\right\|}_{2}},\;\;\;\;{\tilde{z}}_{i}^{WV}=\frac{z_{i}^{WV}}{{\left\|z_{i}^{WV}\right\|}_{2}}.$$

Supervised contrastive learning is applied to encourage embeddings from the same class to form compact clusters while separating embeddings from different classes [21]. In addition, view-level alignment encourages the FFT and wavelet embeddings of the same input segment to have consistent directions as follows:

(13) $$L_{view}=-\frac{1}{B}\sum_{i=1}^{B}\langle {\tilde{z}}_{i}^{FFT},{\tilde{z}}_{i}^{WV}\;\rangle .$$

Cross-spectrum alignment is used to preserve consistency between the global spectral signature of the raw input signal and the spectral structure derived from the wavelet magnitude map. The spectral signatures are computed as follows:

(14) $$s_{i}^{x}=\frac{\log\left(1+\left|RFFT(x_{i})\right|\right)}{{\left\|\log\left(1+\left|RFFT(x_{i})\right|\right)\right\|}_{2}},$$

(15) $$s_{i}^{WV}=\frac{\log\left(1+\bar{\left|RFFT(A_{d,i})\right|}\right)}{{\left\|\log\left(1+\bar{\left|RFFT(A_{d,i})\right|}\right)\right\|}_{2}},$$

where $A_{d,i}$ denotes the wavelet energy map of the i-th sample after aggregation over the filter and dilation dimensions.

The cross-spectrum alignment loss is defined using the inner product between the two normalized spectral signatures.

(16) $$L_{csa}=-\frac{1}{B}\sum_{i=1}^{B}\langle s_{i}^{x},s_{i}^{WV}\;\rangle .$$

The overall Stage II objective combines the supervised contrastive loss, view-level alignment loss, and cross-spectrum alignment loss as follows:

(17) $$L=L_{sup}+{\lambda_{view}L}_{view}+\lambda_{csa}L_{csa},$$

where $L_{sup}$ denotes the supervised contrastive loss, $L_{view}$ denotes the view-level alignment loss, and $L_{csa}$ denotes the cross-spectrum alignment loss. During this stage, only a limited subset of the later layers in each feature extractor is fine-tuned, while the earlier layers remain frozen. After Stage II, the projection heads are removed, and the aligned features $h_{FFT}$ and $h_{WV}$ are used as inputs to the fusion block.

### 3.5. Feature Fusion and Classification

In Stage III, the LFT and LWT feature extractors trained in Stage I and aligned in Stage II are reused with all their parameters frozen. Backpropagation is blocked through the extractors, and only the feature fusion block and final classifier are optimized. This allows Stage III to model interactions between the aligned spectral and wavelet representations while preserving the feature structures learned in the preceding stages.

The two feature vectors are first projected into a common latent dimension.

(18) $${\tilde{h}}_{i}^{FFT}=W_{p}^{FFT}h_{i}^{FFT}+b_{p}^{FFT},\;\;\;{\tilde{h}}_{i}^{WV}=W_{p}^{WV}h_{i}^{WV}+b_{p}^{WV}.$$

Cross-gating is then applied so that each branch is modulated by information from the other branch:

(19) $$u_{i}^{FFT}=\sigma \left(W_{g}^{FFT}{\tilde{h}}_{i}^{WV}+b_{g}^{FFT}\right),$$

(20) $$u_{i}^{WV}=\sigma \left(W_{g}^{WV}{\tilde{h}}_{i}^{FFT}+b_{g}^{WV}\right),$$

where σ(⋅) denotes the sigmoid function.

The gates are applied by element-wise multiplication,

(21) $${\bar{h}}_{i}^{FFT}=u_{i}^{FFT}⊙{\tilde{h}}_{i}^{FFT},\;\;\;\;{\bar{h}}_{i}^{WV}=u_{i}^{WV}⊙{\tilde{h}}_{i}^{WV},$$

where ⊙ denotes element-wise multiplication.

To capture cross-view interactions and differences, the interaction and discrepancy terms are computed as follows:

(22) $$c_{i}={\tilde{h}}_{i}^{FFT}⊙{\tilde{h}}_{i}^{WV}{,\;\;d}_{i}=\left|{\tilde{h}}_{i}^{FFT}-{\tilde{h}}_{i}^{WV}\right|.$$

The final fused representation is formed by concatenating the gated features, interaction term, and discrepancy term.

(23) $$f_{i}=\left[{\bar{h}}_{i}^{FFT};{\bar{h}}_{i}^{WV};c_{i};d_{i}\right].$$

The fused representation is passed through fully connected layers and a 10-class softmax classifier,

(24) $${\hat{y}}_{i}=softmax\left(W_{c}\phi \left(BN\left(f_{i}\right)\right)+b_{c}\right),$$

where BN(⋅) denotes batch normalization, ϕ(⋅) denotes the ReLU activation, and $W_{c}$ and $b_{c}$ are classifier parameters.

The classifier is trained using the multiclass cross-entropy loss,

(25) $$L_{cls}=-\frac{1}{B}\sum_{i=1}^{B}\sum_{c=1}^{C}y_{i,c}\log{\hat{y}}_{i,c},$$

where B is the mini-batch size and C=10 is the number of classes. Because only the fusion block and classifier are updated in Stage III, the final classifier learns to integrate the aligned FFT and wavelet representations while preserving the feature structures obtained in the previous stages.

## 4. Experimental Results and Discussion

### 4.1. Experimental Setup and Model Efficiency

Experiments were conducted using the current waveform dataset described in Section 2. All models were trained and evaluated using the same measurement-session-level group-wise split, with test sessions excluded from training and validation. For fair comparison, all baselines were derived from the same segmented waveforms and used the same segment-wise normalization and data split; the transform-based baselines applied their designated fixed transforms to these waveform segments.

The proposed framework followed the three-stage training procedure described in Section 3. The LFT and LWT branches were independently trained in Stage I, partially fine-tuned using the representation-alignment objective in Stage II, and frozen in Stage III while only the fusion block and final classifier were optimized. Performance was evaluated using accuracy, macro F1-score, and macro recall. The results are reported as the mean ± standard deviation over five independent runs with different random seeds.

Table 3 summarizes the model complexity, model-only inference latency, and calibration metrics of the proposed variants. Ours-FFT-Only and Ours-WV-Only contain 0.35 M and 0.25 M parameters, respectively, whereas Ours-Full contains 1.06 M parameters due to the dual front-ends and feature fusion components. Ours-Full achieved model-only inference latencies of 8.70 ms at batch size 1 and 15.73 ms at batch size 64. Although processing and fusing both feature streams increased computation, Ours-Full showed the lowest expected calibration error (ECE) and Brier score among the proposed variants, suggesting more consistent probability estimates [22,23].

The complexity, latency, and calibration metrics provide complementary perspectives on the practical characteristics of the proposed framework. The parameter count and model-only latency quantify the computational burden of each variant, whereas ECE and the Brier score assess the reliability of the predicted class probabilities. The lower calibration errors of Ours-Full suggest that its confidence estimates are more consistent with the observed classification outcomes, which may be useful when model probabilities are incorporated into threshold-based alarm decisions. However, the reported latency includes only model inference and excludes signal acquisition, window construction, data transfer, and trip actuation. It should therefore be interpreted as model execution time rather than the complete sensing-to-trip response time, which remains to be evaluated in future deployment studies.

### 4.2. Ablation Analysis

Table 4 presents the ablation results for the proposed framework. The LFT and LWT front-ends were kept identical across all variants, while the Stage II alignment losses and the Stage III fusion components were removed or simplified. Thus, the observed differences mainly reflect changes in the representation-alignment and feature fusion configurations.

Ours-Concat directly concatenates the FFT and wavelet features without Stage II alignment or interaction-aware fusion and achieved lower accuracy, macro F1-score, and macro recall than Ours-Full. Ours-Full without Stage II achieved 99.88% accuracy, 99.89% macro F1-score, and 99.89% macro recall. This result is consistent with the intended role of Stage II in organizing the independently learned FFT- and wavelet-based representations before fusion.

The variants without SupCon, cross-spectrum alignment, or view-alignment loss also showed slightly lower mean performance than Ours-Full. SupCon promotes class-level separability, cross-spectrum alignment promotes coherence between the raw input spectral and wavelet-derived representations, and view-level alignment encourages consistency between the FFT- and wavelet-based representations generated from the same input segment. However, the absolute differences were small and comparable to the observed run-to-run variation. Therefore, these results are interpreted as supporting the joint use of the three alignment objectives rather than demonstrating large standalone gains.

The variant without gating and multiplicative interaction achieved 99.78% accuracy, 99.80% macro F1-score, and 99.83% macro recall. This result is consistent with the intended role of Stage III in combining condition-adaptive feature weighting and cross-view interactions. Overall, Ours-Full achieved the highest mean performance, with 99.92% accuracy, 99.92% macro F1-score, and 99.93% macro recall. Taken together, the ablation results support the overall staged configuration of independent front-end learning, representation alignment, and subsequent feature fusion.

### 4.3. Comparison with Baseline Methods

Table 5 compares the proposed model with representative baseline methods. The evaluated methods include conventional machine learning models, raw-waveform deep learning models, fixed-transform-based models, learnable single-front-end variants, and the proposed full model. All methods were evaluated using the same measurement-session-level group-wise split to ensure a consistent comparison. Recent studies have investigated machine learning and deep learning-based arc-fault detection using handcrafted features, current waveform reconstruction, arc-model-based signal generation, and convolutional neural networks [24,25,26,27]. Other studies have further improved detection performance using lightweight neural networks, CNN–Transformer structures, cost-sensitive feature extraction, multimodal feature fusion, and hybrid time–frequency learning strategies [28,29,30,31,32,33,34].

The conventional machine learning baselines showed the lowest performance among the compared methods. SVM achieved 89.25% accuracy, whereas Random Forest achieved 91.22% accuracy. These results indicate that the conventional machine learning baselines were less effective at capturing the diverse waveform variations caused by different loads and arc-fault states.

Raw-waveform deep learning models improved the results compared with the conventional machine learning baselines. The 1D-CNN and RNN models achieved accuracy values near 92%, while LSTM and ResNet1D achieved 95.54% and 95.62% accuracy, respectively. TCN provided the strongest performance among the raw-waveform baselines, achieving 97.77% accuracy, 97.77% macro F1-score, and 97.79% macro recall. These results indicate that residual and temporal convolutional architectures can effectively capture temporal patterns directly from raw current waveforms. Nevertheless, TCN remained below the fixed FFT-based model and the proposed learnable front-end variants, suggesting that explicit spectral information and task-adaptive time–frequency representation learning provide additional benefits for arc-fault classification [35].

The fixed-transform-based models showed substantially higher performance than the conventional machine learning baselines and were competitive with the stronger raw-waveform models. Fixed-(FFT + CNN) achieved 98.14% accuracy and 98.31% macro recall, outperforming all evaluated raw-waveform baselines. Fixed-(WV + CNN) achieved 97.32% accuracy and 97.42% macro recall, which were slightly lower than the corresponding results of TCN. These results indicate that spectral and transient time–frequency cues are useful for distinguishing arc-fault patterns, although the relative effectiveness of a fixed transform depends on the selected representation and downstream architecture. Recent time–frequency image, recurrence-plot, wavelet-reconstruction, and data-enhancement-based approaches also show that transforming current signals into discriminative time–frequency representations can improve arc-fault classification under varying operating conditions [36,37,38,39]. However, both fixed-transform models were outperformed by the corresponding learnable front-end variants, suggesting that predefined transform resolutions and filter structures may not fully adapt to load-dependent waveform variations.

Ours-FFT-Only achieved 99.58% accuracy, 99.60% macro F1-score, and 99.63% macro recall, outperforming the fixed FFT-based baseline. This result indicates that the learnable spectral front-end can emphasize task-relevant frequency components more effectively than a fixed FFT representation. Ours-WV-Only also achieved higher performance than the fixed wavelet-based baseline, with 99.16% accuracy, 99.20% macro F1-score, and 99.23% macro recall. This result is consistent with the intended role of the learnable wavelet front-end in capturing localized transient patterns from high-speed current waveforms.

Ours-Full achieved the highest performance across all metrics, with 99.92% accuracy, 99.92% macro F1-score, and 99.93% macro recall. Compared with Ours-FFT-Only and Ours-WV-Only, the full model further improved performance by integrating global spectral information and localized transient information. The improvement over the single-front-end variants is consistent with the complementary roles of the two representations and with the overall trends observed in Table 4.

### 4.4. Class-Wise Result Analysis

**Figure 4:**
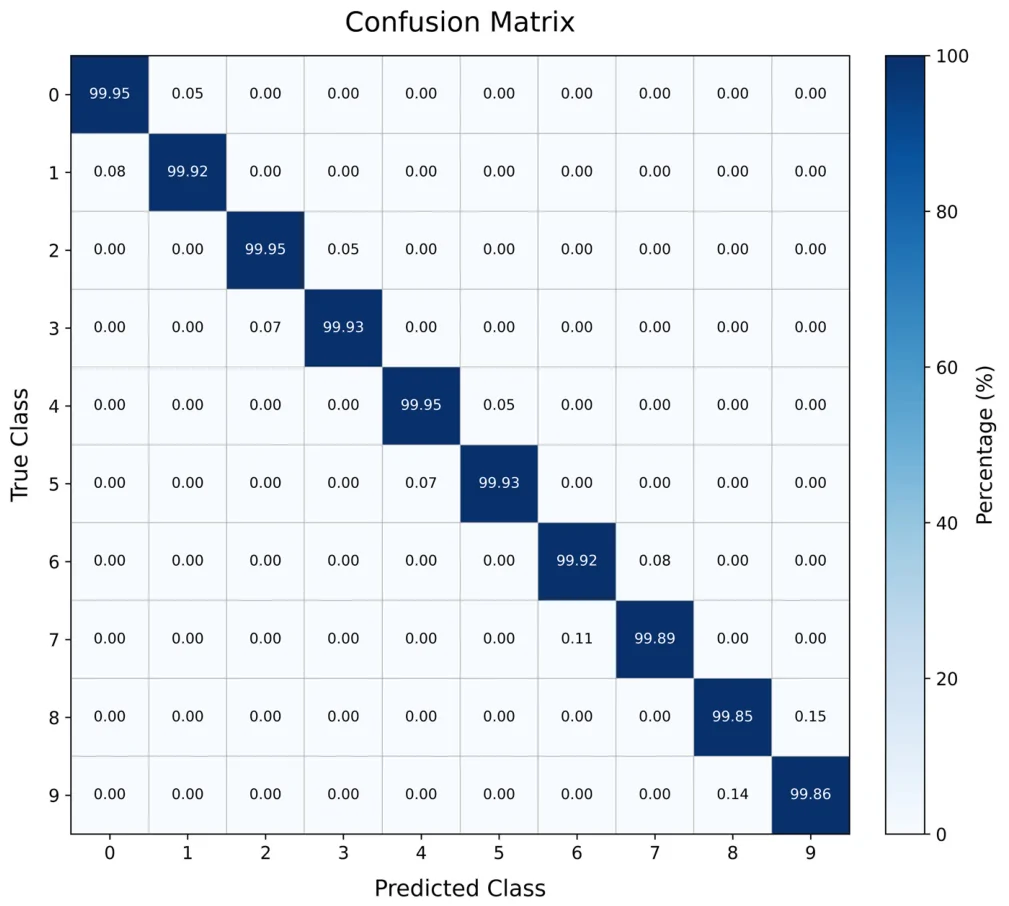
Row-normalized confusion matrix (%) of Ours-Full on the held-out test set.

Figure 4 presents the row-normalized confusion matrix of Ours-Full on the test set. The class indices 0–9 correspond to the load-state definitions listed in Table 1 and to the waveform categories presented in Figure 2. Each row represents a true load-state class, each column represents a predicted class, and each cell reports the percentage of samples from the corresponding true class assigned to the predicted class. Accordingly, the values in each row sum to 100%. Most values are concentrated along the diagonal, indicating that Ours-Full consistently distinguishes the 10 load-state classes. The row-normalized presentation further shows that high classification performance is maintained across classes with different numbers of test samples. This result is consistent with the aggregate performance of Ours-Full reported in Table 5.

The confusion matrix also indicates stable discrimination across both normal and arc-fault conditions for the evaluated load types. Figure 4 reports the confusion matrix obtained from the test run whose accuracy was closest to the five-run mean. Although the ventilation fan, heating lamp, and incandescent lamp waveforms shown in Figure 2 exhibit similar overall shapes, the proposed framework does not rely solely on their visual time-domain characteristics or on a single-domain representation. Instead, the learnable FFT and learnable wavelet front-ends first learn complementary representations from the same current waveform. Stage II then progressively refines these learned representations through supervised alignment objectives, encouraging samples belonging to the same load-state class to become more compact while increasing the separation between different classes in the learned representation space. Finally, Stage III combines these complementary representations through the feature fusion framework to further improve class discrimination. Consequently, initially similar current waveforms are progressively transformed into more discriminative feature representations, enabling reliable separation of visually similar load-state classes. The limited percentages of confusion among the evaluated classes support the effectiveness of this progressive representation-learning process.

Taken together, the results in Table 3, Table 4 and Table 5 and Figure 4 show that the proposed framework improves classification performance while maintaining compact model complexity and low model-only inference latency. The ablation analysis supports the overall staged design, while the baseline comparison indicates the advantage of learnable time–frequency representations over fixed transforms and raw-waveform models. These results support the effectiveness of the proposed progressive framework for robust arc-fault classification across unseen measurement sessions within the evaluated load categories and operating conditions.

### 4.5. ROC and AUC Analysis

**Figure 5:**
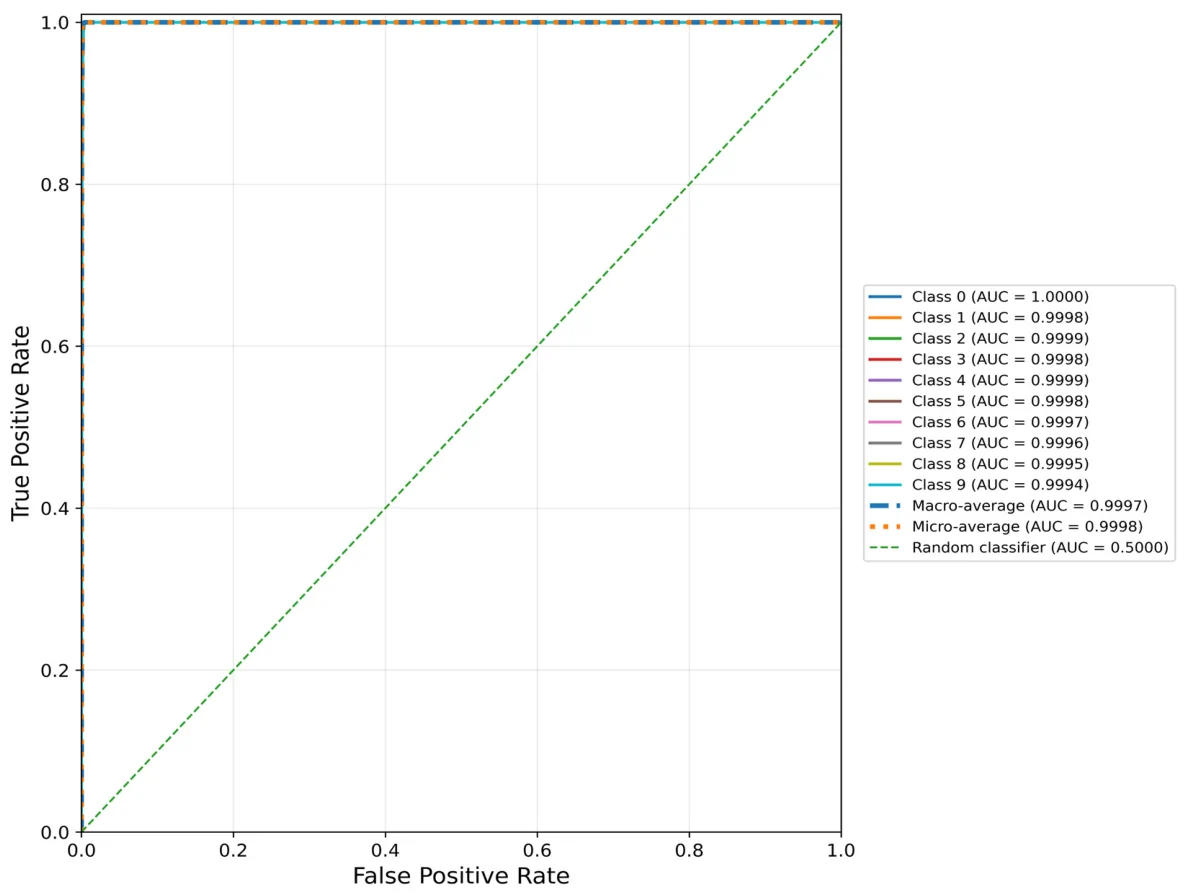
One-versus-rest ROC curves of Ours-Full on the held-out test set.

Figure 5 presents the one-versus-rest receiver operating characteristic (ROC) curves of Ours-Full for the 10 load-state classes on the held-out test set. Figure 5 reports the ROC curves obtained from the same representative test run used for Figure 4. For each class, the corresponding class was treated as positive, while the remaining nine classes were treated as negative. The curves were calculated using the predicted class probabilities across different decision thresholds. The class-wise AUC values ranged from 0.9994 to 1.0000, while the macro-average and micro-average AUC values were 0.9997 and 0.9998, respectively. Consistent with the uniformly high class-wise AUC values, the corresponding ROC curves were nearly superimposed near the upper-left corner of the plot, indicating uniformly strong discrimination across all evaluated load-state classes. These results complement the accuracy, macro F1-score, macro recall, and row-normalized confusion matrix by providing a threshold-independent evaluation of class-wise discrimination performance.

## 5. Conclusions

This paper presented a progressive three-stage time–frequency learning framework for arc-fault detection using high-speed current waveforms acquired from a low-voltage smart-farm distribution system. In Stage I, the proposed framework learns complementary global spectral and local transient representations using FFT-based and wavelet-based learnable front-ends. In Stage II, the two representation spaces are structured using supervised contrastive learning with view-level and cross-spectrum alignment. In Stage III, the aligned feature extractors are kept frozen, while only the feature fusion block and final classifier are optimized for 10-class classification.

The experimental results demonstrated that the proposed framework achieved 99.92% accuracy and 99.93% macro recall for the 10-class load-state classification task under a measurement-session-level group-wise split. The ablation results showed that the complete configuration achieved the highest mean performance among the evaluated variants, supporting the overall staged design of the proposed framework. The model also maintained compact complexity with 1.06 M parameters and achieved 8.70 ms model-only inference latency, indicating the feasibility of millisecond-level neural network decision-making.

Future work will focus on extending the validation to additional farm sites, broader load configurations, and masking-load conditions, and will further examine complete sensing-to-trip system evaluation for practical deployment.

## Figures and Tables

**Table 1:** Class-wise distribution of segmented waveform samples across the training, validation, and test sets.

Class	Label	Training	Validation	Test
0	Ventilation fan-normal	38,220	2940	7840
1	Ventilation fan-arc	41,650	4410	15,680
2	Fluorescent lamp-normal	33,810	3430	11,760
3	Fluorescent lamp-arc	49,000	4900	14,700
4	Heating lamp-normal	34,300	4900	9800
5	Heating lamp-arc	33,810	3920	11,270
6	Incandescent lamp-normal	35,770	3920	9310
7	Incandescent lamp-arc	33,810	4900	10,290
8	LED lamp-normal	38,710	4410	5880
9	LED lamp-arc	36,260	4410	8330
Total		375,340	42,140	104,860

**Table 2:** Dataset construction and segmentation settings.

Item	Value
Sampling rate	1 MS/s
Raw signal duration used for segmentation	Approximately 0.9 s
Input length	18,000 sampled data points
Window duration	18 ms
Stride	1800 sampled data points
Stride duration	1.8 ms
Overlap ratio	90.0%
Segments per measurement session	490
Total samples	522,340
Total measurement sessions	1066
Number of classes	10
Training samples/sessions	375,340/766
Validation samples/sessions	42,140/86
Test samples/sessions	104,860/214

**Table 3:** Model complexity, inference latency, and calibration-related reliability of proposed model variants.

Model Name	Parameters (M)	Latency (ms), *B* = 1	Latency (ms), *B* = 64	ECE	Brier Score
Ours-FFT-Only	0.35	4.40	4.48	0.006	0.004
Ours-WV-Only	0.25	4.70	12.01	0.009	0.007
Ours-Full	1.06	8.70	15.73	0.002	0.001

**Table 4:** Ablation results for fusion and representation-alignment components.

Model Name	Accuracy (%)	Macro F1-Score(%)	Macro Recall (%)
Ours-Concat	99.65 ± 0.06	99.69 ± 0.05	99.72 ± 0.05
Ours-Full (w/o Stage II)	99.88 ± 0.03	99.89 ± 0.03	99.89 ± 0.04
Ours-Full (w/o SupCon)	99.84 ± 0.04	99.85 ± 0.04	99.88 ± 0.03
Ours-Full (w/o Cross-spectrum Alignment)	99.83 ± 0.05	99.84 ± 0.04	99.87 ± 0.04
Ours-Full (w/o View-alignment Loss)	99.82 ± 0.05	99.83 ± 0.05	99.86 ± 0.04
Ours-Full (w/o Gating and Multiplicative Interaction)	99.78 ± 0.06	99.80 ± 0.05	99.83 ± 0.05
**Ours-Full**	**99.92 ± 0.02**	**99.92 ± 0.02**	**99.93 ± 0.02**

**Table 5:** Classification performance comparison with baseline methods.

Model Name	Accuracy (%)	Macro F1-Score (%)	Macro Recall (%)
SVM	89.25 ± 0.28	89.31 ± 0.26	89.33 ± 0.27
Random Forest	91.22 ± 0.21	91.30 ± 0.19	91.34 ± 0.20
1D-CNN	92.67 ± 0.14	92.72 ± 0.13	92.75 ± 0.12
RNN	92.41 ± 0.17	92.43 ± 0.15	92.44 ± 0.16
LSTM	95.54 ± 0.18	95.57 ± 0.17	95.61 ± 0.17
ResNet1D	95.62 ± 0.16	95.63 ± 0.16	95.63 ± 0.15
TCN	97.77 ± 0.13	97.77 ± 0.13	97.79 ± 0.12
Fixed-(FFT + CNN)	98.14 ± 0.09	98.22 ± 0.08	98.31 ± 0.08
Fixed-(WV + CNN)	97.32 ± 0.11	97.40 ± 0.10	97.42 ± 0.10
Ours-FFT-Only	99.58 ± 0.05	99.60 ± 0.05	99.63 ± 0.04
Ours-WV-Only	99.16 ± 0.07	99.20 ± 0.06	99.23 ± 0.06
Ours-Full	99.92 ± 0.02	99.92 ± 0.02	99.93 ± 0.02

## Data Availability

The data that support the findings of this study are not publicly available due to project and data-owner restrictions but are available from the corresponding author upon reasonable request and with appropriate permission.

## Abbreviations

The following abbreviations are used in this manuscript:

FFT	Fast Fourier Transform
LFT	Learnable Fourier Transform
LWT	Learnable Wavelet Transform
WV	Wavelet
CNN	Convolutional Neural Network
ECE	Expected Calibration Error
RFFT	Real-Valued Fast Fourier Transform
SupCon	Supervised Contrastive Learning
